# miR-128-3p inhibits intramuscular adipocytes differentiation in chickens by downregulating *FDPS*

**DOI:** 10.1186/s12864-023-09649-y

**Published:** 2023-09-12

**Authors:** Shuaipeng Zhu, Binbin Zhang, Tingqi Zhu, Dongxue Wang, Cong Liu, Yixuan Liu, Yuehua He, Wenjie Liang, Wenting Li, Ruili Han, Donghua Li, Fengbin Yan, Yadong Tian, Guoxi Li, Xiangtao Kang, Zhuanjian Li, Ruirui Jiang, Guirong Sun

**Affiliations:** 1 College of Animal Science and Technology, Henan Agricultural University, Zhengzhou, 450046 P.R. China; 2 The Shennong Seed Industry Laboratory, Zhengzhou, 450002 China

**Keywords:** Chicken, Intramuscular adipocyte, RNA-seq, MiR-128-3p, *FDPS*, Adipogenesis

## Abstract

**Background:**

Intramuscular fat (IMF) content is the major indicator for evaluating chicken meat quality due to its positive correlation with tenderness, juiciness, and flavor. An increasing number of studies are focusing on the functions of microRNAs (miRNAs) in intramuscular adipocyte differentiation. However, little is known about the association of miR-128-3p with intramuscular adipocyte differentiation. Our previous RNA-seq results indicated that miR-128-3p was differentially expressed at different periods in chicken intramuscular adipocytes, revealing a possible association with intramuscular adipogenesis. The purpose of this research was to investigate the biological functions and regulatory mechanism of miR-128-3p in chicken intramuscular adipogenesis.

**Results:**

The results of a series of assays confirmed that miR-128-3p could promote the proliferation and inhibit the differentiation of intramuscular adipocytes. A total of 223 and 1,050 differentially expressed genes (DEGs) were identified in the mimic treatment group and inhibitor treatment group, respectively, compared with the control group. Functional enrichment analysis revealed that the DEGs were involved in lipid metabolism-related pathways, such as the MAPK and TGF-β signaling pathways. Furthermore, target gene prediction analysis showed that miR-128-3p can target many of the DEGs, such as *FDPS*, *GGT5*, *TMEM37*, and *ASL2.* The luciferase assay results showed that miR-128-3p targeted the 3’ UTR of *FDPS*. The results of subsequent functional assays demonstrated that miR-128-3p acted as an inhibitor of intramuscular adipocyte differentiation by targeting *FDPS*.

**Conclusion:**

miR-128-3p inhibits chicken intramuscular adipocyte differentiation by downregulating *FDPS.* Our findings provide a theoretical basis for the study of lipid metabolism and reveal a potential target for molecular breeding to improve meat quality.

**Supplementary Information:**

The online version contains supplementary material available at 10.1186/s12864-023-09649-y.

## Background

Poultry meat is an important animal origin food source which can provide essential nutrients [1]. The nutritional value of poultry meat has recently received tremendous attention [2]. A Chinese local chicken breed, Gushi chicken is favored by most consumers for its delicious and uniquely flavored meat. Intramuscular fat (IMF) content is an important indicator affecting many aspects of poultry meat quality, including flavor, tenderness, and taste [3, 4]. IMF deposition depends mainly on hypertrophy and hyperplasia of adipocytes [5–7]. IMF deposition studies have been reported in major livestock and poultry species, such as cattle [8], sheep [9], and chickens [10]. Our previous studies showed significant differences in the density and size of lipid droplets in breast muscle between the early and late stages of egg laying as well as between groups [11]. Therefore, an in-depth study of adipocyte proliferation and differentiation is particularly important.

MicroRNAs (miRNAs) are short noncoding RNAs of approximately 22 nucleotides that negatively regulate gene expression at the posttranscriptional level by recognizing the 3′ UTR of a target mRNA [12–14]. Studies have shown that miRNAs are involved in regulating various biological processes, including lipid metabolism, adipogenesis, cell proliferation and cell differentiation [15–17]. For instance, miR-33a inhibits the differentiation of bovine preadipocytes through the IRS2-Akt pathway [18]. The miR-429-3p/LPIN1 axis promotes chicken abdominal fat deposition via the *PPARγ* pathway [19], while miR-130a suppresses adipogenic differentiation of BMSCs by targeting *PPARγ* [20]. Recent studies have also suggested that miR-128-3p may be involved in the regulation of fat deposition in humans [21], mice [22] and chickens [23]. For example, miR-128-3p may target *IRS-1*, *FOXO1*, *SREBP-1c/2* and *ChREBP*, which are functionally involved in lipid and lipoprotein metabolism as well as insulin signaling [24]. In addition, miR-128-3p has a targeting relationship with the adipose differentiation marker gene *PPARγ* [23]. Moreover, our previous transcriptome analysis found significant differences in the expression of miR-128-3p before and after intramuscular adipocyte differentiation, and we thus speculated that miR-128-3p may be a core miRNA regulating intramuscular adipose differentiation [25]. However, the regulatory mechanism of miR-128-3p in chicken intramuscular adipogenesis remains unclear.

In this study, we verified the effect of miR-128-3p in intramuscular adipocytes of Gushi chickens and compared the dynamic changes in lipid metabolism after miR-128-3p overexpression and interference. We identified several key mRNAs that play a role in the regulation of lipid metabolism pathways via miR-128-3p using transcriptome data and functional prediction. In addition, the results of our cell functional assays confirmed that miR-128-3p regulates the differentiation of intramuscular adipocytes in chickens. Our findings also provide valuable prospects for clarifying the process of IMF deposition in chickens and further contribute to the improvement of meat quality in farm animals.

## Results

### miR-128-3p promotes the proliferation of chicken intramuscular adipocytes

**Fig. 1:**
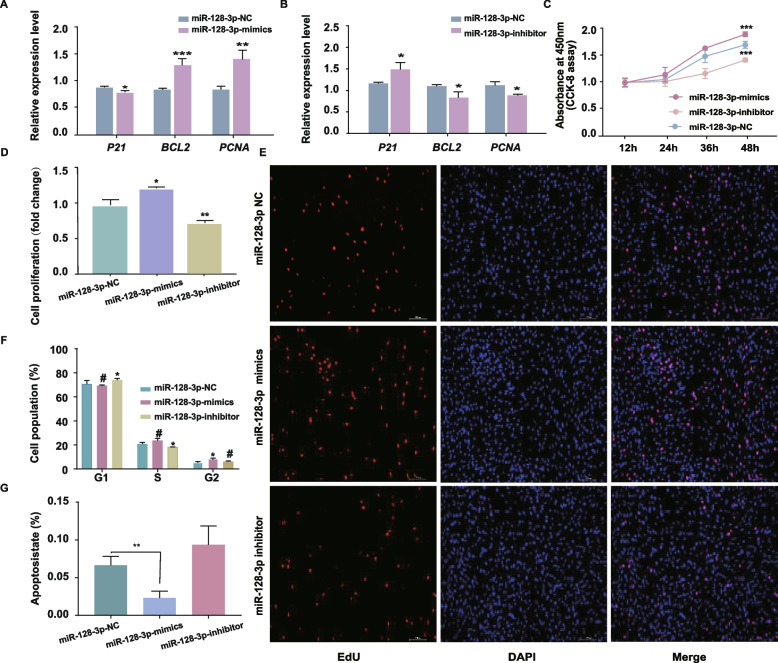
miR-128-3p promoted the proliferation of intramuscular adipocytes. **A**,** B** Relative mRNA levels of cell proliferation-related genes (*P21*, *BCL2*, *PCNA*) after miR-128-3p overexpression or interference. **C** Cell growth curves determined by a CCK-8 assay at 12 h, 24 h, 36 h, and 48 h following miR-128-3p overexpression or interference. **D** Proliferation state of preadipocytes, as assessed by an EdU incorporation assay, after miR-128-3p overexpression or interference. **E** Cell cycle analysis via flow cytometry after miR-128-3p overexpression or interference. **F** Apoptosis assay via flow cytometry after miR-128-3p overexpression or interference. The results are shown as the mean ± S.E.M. values, and the data are representative of at least three independent assays. Independent samples t tests were used to analyze the significance of differences between groups (.^#^ *P* > 0.05, * *P* < 0.05; ** *P* < 0.01, *** *P* < 0.001)

Intramuscular adipocytes were transfected with the miR-128-3p mimic or inhibitor or with the negative control (NC) for 24 h. RT‒qPCR analysis showed that the miR-128-3p expression level was significantly increased in intramuscular adipocytes transfected with the miR-128-3p mimic compared to the NC (*P* < 0.001) (Fig. S1A) and was significantly decreased by transfection of the miR-128-3p inhibitor compared to the NC (*P* < 0.001) (Fig. S1B). These results indicated that the transfection experiment was effective and guaranteed the reliability of the data obtained in follow-up investigations. In chicken intramuscular adipocytes, overexpression of miR-128-3p significantly increased the expression of the marker genes *BCL2* and *PCNA*, which promote cell proliferation, and significantly decreased the expression of *P21*, which inhibits cell proliferation (*P* < 0.05) (Fig. 1A). In contrast, miR-128-3p inhibition significantly decreased the expression of *BCL2* and *PCNA* and significantly increased the expression of *P21* (*P* < 0.05) (Fig. 1B). The results of the CCK-8 assay showed that overexpression of miR-128-3p promoted the proliferation of intramuscular adipocytes, whereas inhibition of miR-128-3p inhibited the proliferation of intramuscular adipocytes (Fig. 1C). The results of the EdU incorporation assay showed that the proportion of EdU-positive cells increased and decreased correspondingly after miR-128-3p overexpression and interference, respectively (Fig. 1D, E). To further confirm the role of miR-128-3p in the proliferation of chicken intramuscular adipocytes, we conducted overexpression experiments. Upon miR-128-3p overexpression, the numbers of cells in S phase and G2 phase increased significantly (*P* < 0.05), while the number of cells in G1 phase decreased (Fig. 1F, Fig. S2). The results of the apoptosis assay showed that the apoptosis rate decreased dramatically after transfection with the miR-128-3p mimic (*P* < 0.05) (Fig. 1G, Fig. S3). The opposite effects were observed when the expression of miR-128-3p was downregulated (*P* < 0.05). All above indicate that miR-128-3p may promote the proliferation of chicken intramuscular adipocytes.

### miR-128-3p inhibits the differentiation of chicken intramuscular adipocytes

**Fig. 2:**
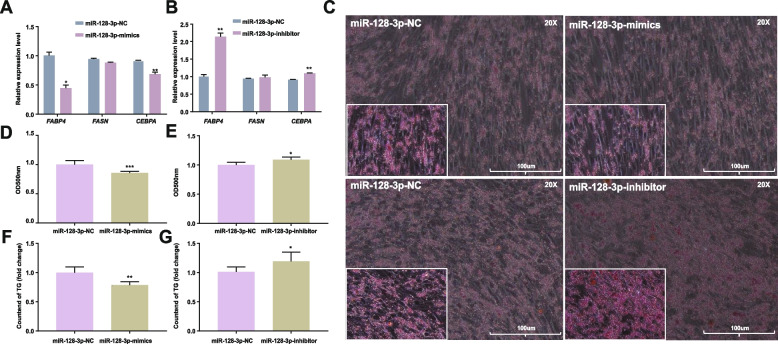
miR-128-3p inhibited the differentiation of chicken intramuscular adipocytes. **A**,** B** Relative mRNA levels of cell proliferation-related genes (*FABP4*, *FASN* and *CEBPA*) after miR-128-3p overexpression or interference. **C** Representative images of Oil Red O staining in intramuscular adipocytes transfected with the miR-128-3p mimic, miR-128-3p inhibitor or corresponding NC (this image was acquired with a 20 × objective, and an enlarged image is shown in the bottom left corner of the image). **D**, **E** Semiquantitative assessment of Oil Red O absorbance at 450 nm. **F**, **G** The triglyceride content was determined by measurement of the absorbance at 500 nm after miR-128-3p overexpression or interference

The expression of adipogenic genes (*FABP4*, *FASN*, and *CEBPA*) was measured in chicken intramuscular adipocytes following transfection. The expression of the adipocyte differentiation marker genes *FABP4* and *CEBPA*, which promote fat deposition, was substantially decreased and increased after miR-128-3p overexpression and interference, respectively (*P* < 0.05) (Fig. 2A, B). We next sought to further investigate the effect of adipocyte differentiation on lipid droplet accumulation and intracellular triglyceride (TG) content in chickens. Oil Red O staining demonstrated that overexpression of miR-128-3p greatly decreased lipid accumulation in intramuscular adipocytes (*P* < 0.05) (Fig. 2C, D) and that miR-128-3p inhibitor transfection had the opposite effect on adipogenesis (*P* < 0.05) (Fig. 2C, E). In addition, the results of the triglyceride assay showed that overexpression of miR-128-3p decreased intracellular triglycerides (*P* < 0.01) (Fig. 2F) but interference with miR-128-3p increased intracellular triglycerides (Fig. 2G). Summarize the above, these data that indicate miR-128-3p has the ability to prevent chicken intramuscular adipocytes from differentiating.

### Transcriptome data analysis and screening of miR-128-3p target genes

**Fig. 3:**
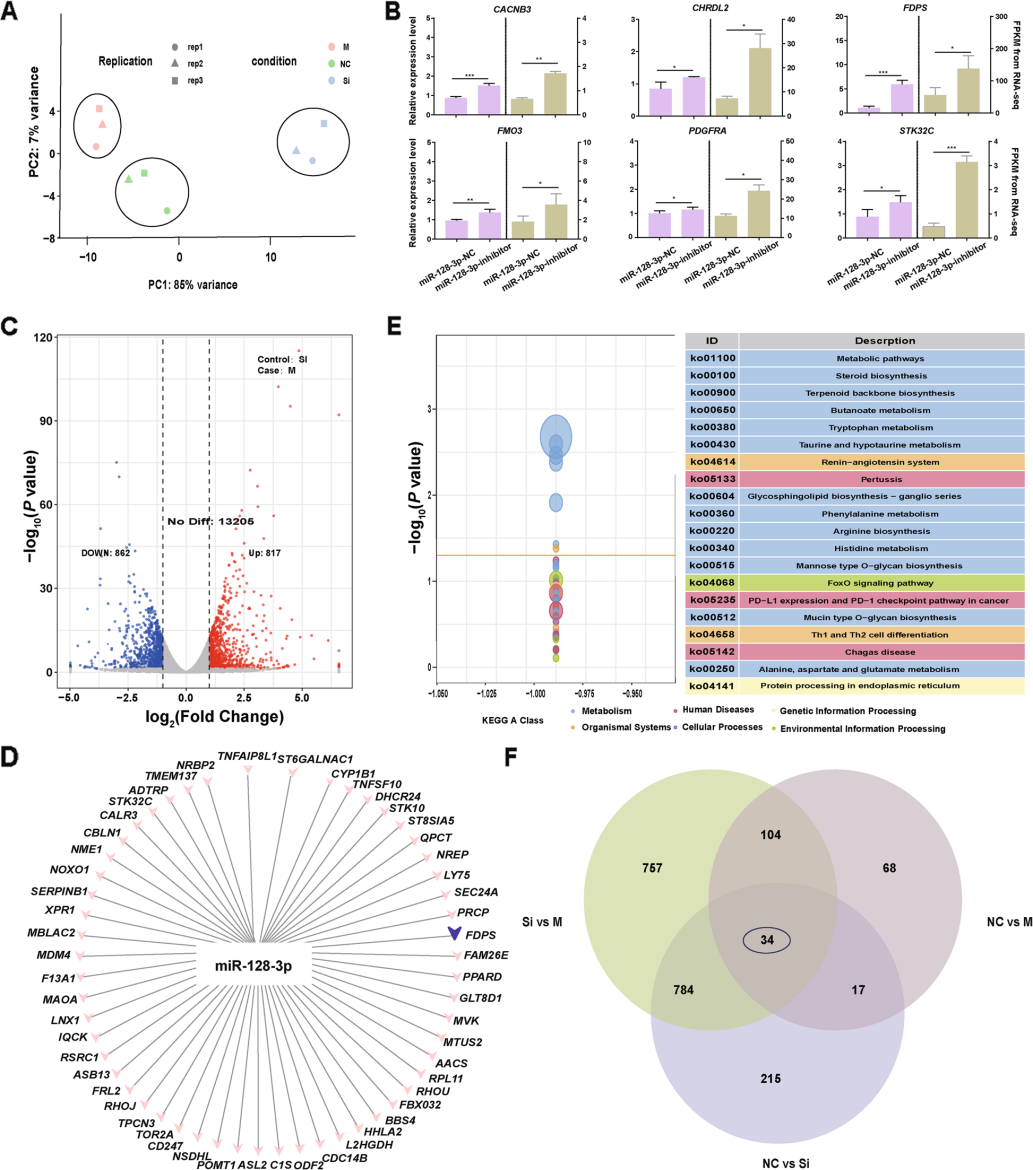
Differential mRNA expression analysis. **A** PCA of the samples. **B** Validation of mRNA sequencing data. **C** Volcano plot of gene expression in the SI *vs*. M comparison. Numbers of upregulated and downregulated differentially expressed mRNAs. The left blue bars represent the numbers of upregulated genes; the orange bars represent the numbers of downregulated genes.** D** Based on the comparison of binding sites in the seed region of miR-128-3p, Cytoscape software was used for network interaction analysis, and the regulatory network of miR-128-3p was mapped. **E** KEGG pathway enrichment analysis of the DEGs in the M *vs*. SI comparison. **F** Venn diagrams of DEGs identified by RNA-seq in the NC *vs*. M, NC *vs*. SI, and SI *vs*. M comparisons

To further explore the gene regulatory pattern associated with miR-128-3p in intramuscular adipocytes and screen for downstream regulated genes, we performed transcriptome sequencing of intramuscular adipocytes after transfection with the miR-128-3p mimic and inhibitor. The clean reads were compared with the reference genome (Gallus_gallus-7.0), and the results showed that the average mapping rate of the clean reads was higher than 97% (Supplementary Table 2–4). The distribution of reads aligning to the genome was determined, and the findings indicated that approximately 85% of the reads were distributed on genes, approximately 15% of the reads were distributed in intergenic regions, and more than 90% of the reads were distributed in exonic regions (Supplementary Table 5). To explore the mRNA changes and biological clustering across groups, we first performed principal component analysis (PCA). PCA for the three groups showed that 92% of the variance could be explained by the first two principal components (Fig. 3A), indicating the diverse mRNA profiles across these three groups. To confirm the reliability of the RNA-seq results, six DEGs (*CNCNB3*, *CHRDL2*, *FDPS*, *FMO3*, *PDGFRA*, and *STK32C*) were randomly selected, and their expression levels were measured by RT‒qPCR (Fig. 3B). As expected, the expression levels of all six candidate genes showed a consistent trend of expression, therefore validating our results.

A total of 1,689 differentially expressed mRNAs, namely, 817 upregulated and 862 downregulated mRNAs, were identified by comparing the overexpression group (M group) with the interference group (SI group) (Fig. 3C). Functional enrichment analysis revealed that the DEGs were involved in lipid metabolism-related pathways, such as the MAPK and TGF-β signaling pathways (Fig. S4C, F). These DEGs, such as *APOA1*, *FDPS*, *MSMO1*, and *HMGCS1*, were identified to potentially regulate chicken preadipocyte differentiation (Supplementary Table 6). Upregulated miRNAs affect fat accumulation by downregulating their target mRNAs. Therefore, we performed network interaction analysis using Cytoscape software and plotted the regulatory network of miR-128-3p. In this network, miR-128-3p can target 56 down regulated DEGs, such as *FDPS*, *GGT5*, *TMEM37*, and *ASL2*, based on the comparison of binding sites in the miR-128-3p seed region (Fig. 3D). Enrichment analysis showed that these potential target genes were mainly involved in metabolic pathways, including steroid biosynthesis and terpenoid backbone and butanoate metabolism (Fig. 3E). To ensure that the selected target genes indeed play a role in IMF deposition, we investigated whether these potential target genes overlapped among the three comparisons (NC *vs.* M, NC *vs.* SI, and SI *vs.* M). The intersection of the overlapping genes indicated that 34 DEGs were potential target genes of miR-128-3p (Fig. 3F, Table S7).

### FDPS as a target gene of miR-128-3p

**Fig. 4:**
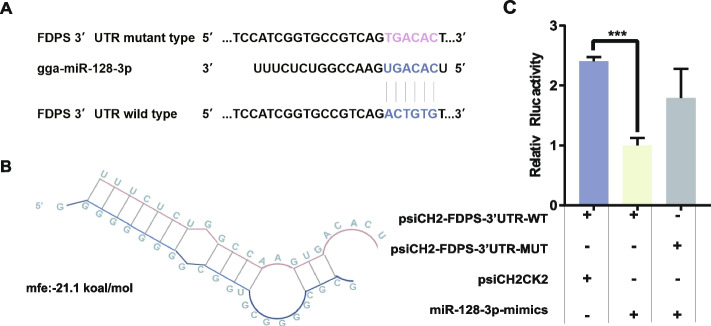
*FDPS* as a target gene of miR-128-3p. **A** The potential miR-128-3p target site in the *FDPS* mRNA 3’UTR was predicted by the RNAhybrid tool. **B** The miR-128-3p binding site in the FDPS mRNA 3’UTR.** C** A dual luciferase reporter assay was performed by cotransfecting plasmids containing the wild-type or mutated FDPS 3’UTR, the psiICH2CK2 plasmid and the miR-128-3p mimic into DF-1 cells. The results are shown as the mean ± S.E.M. values, and the data are representative of at least three independent assays. Independent samples t tests were used to analyze the significance of differences between groups (*** *P* < 0.001)

We predicted the binding capacity of the gene 3'UTR region of miR-128-3p using an online website (https://bibiserv.cebitec.unibielefeld.de/rnahybrid/submission.html/). The results showed that *FDPS* had strong binding ability with both miR-128-3p (Fig. 4A, B). To validate this prediction, we then performed dual luciferase report assay, and the results supported that miR-128-3p mimics can significantly inhibit the luciferase activity of psiCHECK2-*FDPS* 3'UTR-WT. However, it has no effect on psiCHECK2-*FDP*S 3'UTR-MuT (Fig. 4C). It indicated that *FDPS* gene and miR-128-3p were in a targeted binding relationship.

### Interference with FDPS inhibits intramuscular preadipocytes differentiation

**Fig. 5:**
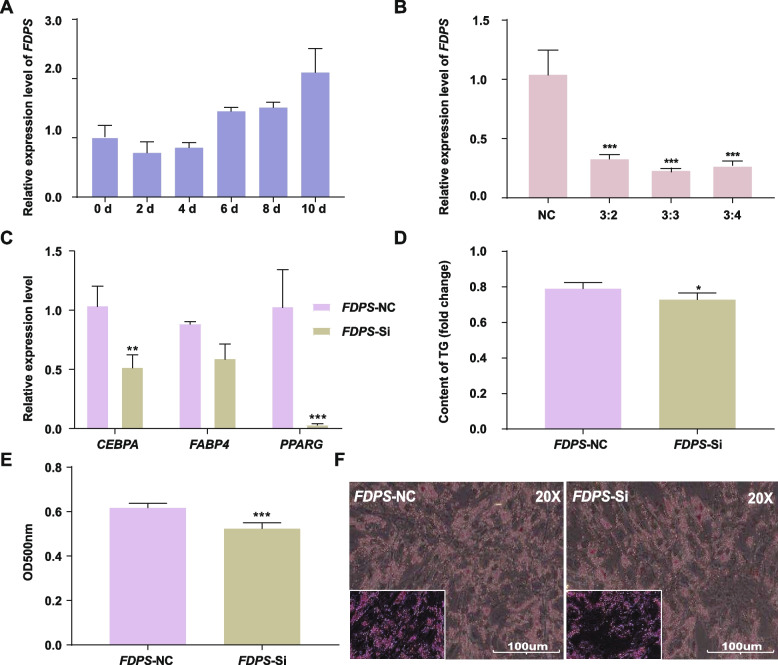
Interference with *FDPS* expression inhibits intramuscular preadipocyte differentiation. **A** mRNA levels of *FDPS* during the differentiation of chicken primary intramuscular adipocytes into mature adipocytes. **B** Relative expression of *FDPS* in intramuscular adipocytes transfected with *FDPS*. **C** Relative mRNA levels of adipocyte differentiation-related genes (*FABP4*, *FASN*, and *CEBPA*) after interference with *FDPS* expression. **D** The triglyceride content was determined by measurement of the absorbance at 500 nm after interference with FDPS expression. **E** Representative images of Oil Red O staining in intramuscular adipocytes transfected with FDPS siRNA or the corresponding NC (this image was acquired with a 20 × objective, and an enlarged image is shown in the bottom left corner of the image.). **F** Semiquantitative assessment of Oil Red O absorbance at 450 nm

To verify whether *FDPS* and miR-128-3p exert opposing regulatory effects on fat deposition, we conducted in vitro experiments to investigate the effect of *FDPS* on intramuscular adipocyte differentiation. During intramuscular preadipocyte differentiation, an increasing trend was observed in the expression of *FDPS* from 0 to 10 d, suggesting that *FDPS* may be related to adipose differentiation as a target gene (Fig. 5A). To further investigate the effect of *FDPS* interference on lipid deposition in intramuscular adipocytes, *FDPS* small interfering RNAs (siRNAs) were transfected into intramuscular adipocytes. Compared with that in the NC cells, the expression level of *FDPS* in transfected intramuscular adipocytes was decreased (Fig. 5B). Quantitative real-time PCR analysis showed that the expression of adipose differentiation marker genes (*CEBPA* and *PPARG*) was significantly reduced (*P* < 0.01), but there was no significant change in the expression level of *FABP4* (Fig. 5C). The results of the triglyceride assay indicated that interfering with *FDPS* gene expression reduced the triglyceride content (Fig. 5D). Moreover, Oil Red O staining showed that interfering with *FDPS* gene expresison reduced lipid accumulation (Fig. 5E, F) (*P* < 0.001). Therefore, these findings further confirm that miR-128-3p can inhibit intramuscular adipocyte differentiation by regulating the expression of *FDPS*.

## Disscussion

Compared with nutritional regulation and feeding management on IMF content, regulation of genetic factors has a more precise influence on IMF accumulation in skeletal muscle [26]. miRNAs can normally bind to the 3’UTRs of their target mRNAs and function as endogenous translational repressors [27]. Thus, miRNAs also play a key role in meat quality and growth performance in livestock and poultry [28, 29]. For example, gga-miRNA-18b-3p can inhibit the differentiation of chicken intramuscular adipocytes by targeting *ACOT13* [25], and miR-223 can inhibit intramuscular adipocyte differentiation by targeting *GPAM* [30]. Our results showed that miR-128-3p promoted intramuscular preadipocyte proliferation to some extent. However, a pair of studies showed that miR-128-3p inhibits hepatocellular carcinoma (HCC) cell proliferation by binding to *PIK3R1* [31] and *CDC6* [32]. Moreover, via repression of *FOXO4* and *MMP9* gene expression, the proliferation of vascular smooth muscle cells was inhibited [33]. These observations reveal that miR-128-3p may play different roles in different cell types. Furthermore, we experimentally determined that miR-128-3p inhibited chicken intramuscular adipocyte differentiation, consistent with the findings of other studies [22, 34, 35].

Identifying the miRNA‒mRNA regulatory network is important for in-depth study of the mechanisms regulating intramuscular adipocyte development. Above, we showed that miR-128-3p was involved in intramuscular adipocyte growth and development. Thus, we performed transcriptome sequencing of intramuscular adipocytes after transfection with the miR-128-3p mimic and inhibitor. In the comparison between the M group and SI group, 817 upregulated and 862 downregulated mRNAs were identified. Functional enrichment analysis revealed that the DEGs in the M group compared with the SI group were involved in the *PPAR* signaling pathway and MAPK signaling pathway, both of which are associated with fat deposition [36, 37]. Several DEGs were regarded as potentially regulating chicken preadipocyte differentiation, for example, *APOA1* [38], *FDPS*, *MSMO1* [39], and *HMGCS1* [40]. More interestingly, miR-128-3p can target many genes that may be involved in lipid deposition, such as *FDPS*, *GGT5*, *TMEM37*, and *ASL2*, based on a comparison of their binding sites with the miR-128-3p seed region [41–45].

miRNAs bind to the 3'UTRs of mRNAs through their core seed region, and current research focuses on miRNAs that regulate lipid deposition through the lipogenic PPAR-α pathway [46], adiponectin receptor pathway, and AMPK pathway [47]. Through target prediction and homology analysis, we found that the *FDPS* 3′UTR can efficiently bind to the miR-128-3p seed region. Our results showed that miR-128-3p can target the *FDPS* gene for binding and that overexpression of miR-128-3p decreased *FDPS* gene expression. Subsequently, we found that downregulation of *FDPS* inhibited intramuscular adipocyte differentiation. RT-qPCR analysis showed that after interference with FDPS expression, the expression of the adipose differentiation marker genes (*CEBPA* and *PPARG*) was significantly reduced, but there was no significant change in the expression level of FABP4. *PPARG* and *CEBPA* play important roles in early adipocyte differentiation, and recent studies have revealed that the *PPARG* and *CEBPA* target genes are colocalized [48]. Therefore, we speculate that there is a clear association among the expression of the *FDPS*, *PPAR*, and *CEBPA* genes, but the associations of these genes with *FABP4* are relatively weak. Studies have shown that genes related to cholesterol biosynthesis, carbohydrate metabolism and fatty acid biosynthesis impact fat development [49]. The mevalonate pathway is a metabolic pathway for the synthesis of isoprenyl pyrophosphate and dimethylallyl pyrophosphate from acetyl-CoA and is the major pathway of de novo cholesterol synthesis in cells [50]. *FDPS* is a branch point enzyme involved in the synthesis of sterols and prenylated cellular metabolites [51, 52]. Therefore, we hypothesized that *FDPS* may affect fat deposition by regulating cholesterol synthesis and affecting acetyl-CoA content. Together, our results indicate that *FDPS* is a functional target of miR-128-3p and can participate in adipogenesis, which is thus regulated by miR-128-3p.

## Conclusion

In conclusion, this study demonstrates that miR-128-3p can inhibit chicken intramuscular adipocyte differentiation by downregulating *FDPS*. Our findings provide a valuable resource for understanding IMF deposition and for explaining the genetic basis of traits related to meat quality in poultry.

## Materials and methods

### Primary intramuscular precursor adipocytes isolation and culture and induced differentiation

The intramuscular adipocytes were from the pectoral muscle tissue of 14-day-old Gushi chicken under sterile conditions [53]. Pectoral muscle tissue was separated using a scalpel and then was digested with collagenase type I (solaibao, Beijing, China) at 37 °C for 60 min. Briefly, the digested cell fraction was filtered sequentially passed through cell strainers (Biologix, Jinan, China) with pore sizes of 70 µm and 45 µm. The cell precipitate was centrifugated at 1000 rpm for 10 min. Subsequently, these cells were maintained in DMEM/F12 medium (BI, Massachusetts, USA), supplemented with 10% fetal bovine serum (BI, Massachusetts, USA), and 1% penicillin/streptomycin (Solarbio)) in an incubator with a 5% CO_2_ atmosphere at 37 °C. After 2 h, the medium was changed. Once upon the confluence of cells reaching 90%, according to the hormone "cocktail method" is used to induce differentiation [54], and the complete medium would be replaced with the differentiation inducing medium (0.5 mM 3-isobutyl-1-methylxanthine (IBMX), 1 uM dexamethasone (Sigma), and 10 g/l insulin (Sigma)).

### Cell transfection

The miRNA mimic/inhibitor and siRNA interference sequences and their corresponding negative controls were purchased from Genechem (Shanghai, China). Cells were transfected with miRNA mimics, inhibitors and *FDPS-*SI with Liposome 2000 reagent (Invitgen, USA) according to the manufacturer's instructions. Transfection was carried out when the cell confluence was 60–70%, and the medium was changed to complete medium after 6 h.

### CCK-8, EdU and cell cycle assay detection of cell proliferation

The intramuscular adipocytes proliferation was detected after 12 h, 24 h, 36 h and 48 h of transfection using a Trans Detect CCK-8 kit (Toyohito, Japan) according to the manufacturer's protocol. 10 μL of CCK-8 solution was added to the cells and incubated at 37 ℃ constant temperature incubator for 2 h. Then, the absorbance value was measured at 450 nm using the microplate reader.

The intramuscular adipocytes was detected using a Cell-Light EdU Apollo567 In Vitro Kit (RiboBio, Guangzhou, China) according to the manufacturer’s protocol after transfection for 24 h. Briefly, 100 mL of 50 mm EdU reagent was added to each well and incubated for 2 h at 37 ℃. After the staining, the camera was photographed in the dark under the fluorescent microscope. Finally, the Imaje J software was used to count the number of new cells.

The intramuscular adipocytes were seeded in 6 well cell culture plates. The cells were transfected with miR-128-3p mimic, miR-128-3p inhibitor, and negative control. After 48 h, Cells were collected, washed with cold PBS, and fixed with 70% ethanol at 20 °C for 6 h. Then the cells were washed with PBS and added for a final mass concentration of 50 mg/mL to incubat for 30 min at 37 °C. Subsequently, 400 μl of 50 μg/mL propidium iodide (PI) solution was added, and the cells were stained in the dark for 30 min at 25 °C. Finally, the cell cycle was observed by flow cytometry (FACS Calibur, BD Biosciences).

### Oil-Red O staining and triglyceride assay detection of cell differentiation

The intramuscular adipocytes samples were washed three times with PBS (Gibco, Carlsbad, CA, USA) and fixed with 4% paraformaldehyde for 30 min [55]. Subsequently, cells were stained with Oil-Red O working solution (Sigma) for 20 min after being washed with PBS. Then the intramuscular adipocytes were photographed by microscope. Lipid droplets were dissolved by Isopropyl alcohol and the absorbance value was calculated at 490 nm using microplate reader.

The intramuscular adipocytes samples were treated with 0.25% trypsin until separation and centrifuged for 3 min at 1000 rpm. After all, TG content in cell homogenate was determined using a triglyceride content detection kit (APPLYGEN, Beijing, China) according to the manufacturer’s instructions. The protein concentrations were measured with the BCA Protein Assay Kit (EpiZyme, Shanghai, China) to normalize the TG content. The absorbance value was calculated at 550 nm using microplate reader.

### Collection of sequencing samples

The transfected miR-128-3p-mimics, miR-128-3p-inhibitor and miR-128-3p-NC were divided into overexpression group (M group), interference group (SI group), and blank treatment group (NC group), respectively. The total RNA was extracted using Trizol reagent (Vazyme, Nanjing, China). The expression of miR-128-3p was detected by fluorescent quantitative PCR. The sample with the highest overexpression efficiency (*n* = 3) and interference efficiency (*n* = 3) was collected, respectively, and sent to Nanjing Parsono Gene Technology Co., Ltd. for transcriptome sequencing.

### Transcriptome data analysis

After the removal of raw reads containing no insertion sequence, over 0.2% of poly-N, and low-quality paired reads, we obtained clean reads. The high-quality data (clean data) were mapped to the reference genome (GRCg7a) using TopHat2's upgraded HISAT2 software [56]. Read count values were aligned to each gene using HTSeq statistics as the gene's original expression quantity [57]. Differential gene expression analysis was conducted using DESeq [58], and differentially expressed genes (DEGs) were defined based on the following criteria: |log_2_ fold change|> 1 and the *P* value < 0.05. The DEGs were subjected to functional annotation and pathway enrichment analysis using the KOBAS server [59].

### RT-qPCR

Total RNA was extracted from tissues and harvested cells using Trizol (Vazyme, Nanjing, China). Reverse transcription of mRNA was performed using a HiScript II Q Select RT SuperMix for qPCR kit (Vazyme, Nanjing, China) according to manufacturer instructions. First step reaction system: 4 µl, 4 × gDNA wiper Mix, Total RNA 1000 ng, added RNase free ddH2O to a total system of 16 µl. The reaction procedure was set to 42 ℃ for 2 min and stored at 4 ℃. Second step reaction system: 4 µl, 5 × HiScript III qRT SuperMix was added to the previous product, resulting in a total system of 20 µl. The reaction procedure was set as 37 ℃ for 15 min and 8 ℃ for 5 s. Finally, the product cDNA was diluted twice for subsequent fluorescence quantitative testing.We designed the PCR primers using Primer Premier 5, and the primer sequences were shown in Supplementary table 1. The ChamQ Universal SYBR qPCR Master Mix kit (Vazyme, Nanjing, China) was used to conduct quantitative real-time PCR (Q-qPCR). The total reaction volume was 10 ul, and consisted of 1 μl cDNA, 0.5 μl reverse and forward primers (per gene), 5 ul SYBR, and 3 ul double-distilled water. The reaction procedure was performed under the following conditions:95 °C for 30 s, followed by 40 cycles at 95 °C for 15 s, and 60 °C for 34 s. Finally, the melting curve is collected at 60–95 °C. Relative expression level was quantified by the 2^−△△Ct^ [60] approach. β-actin and U6 was using as the normalization references for mRNA and miRNA, respectively.

### Dual luciferase reporter assay

The laboratory psiCHECK2 vector was used to construct the psiCHECK2-FDPS-3'UTR-WT and psiCHECK2-FDPS-3'UTR-MuT plasmids. They were transfected with miRNA-128-3p-mimics and psiCHECK2 vector into DF1 cells. After 48 h, the samples were collected, and the fluorescence activity was detected using the Dual-Glo Luciferase Assay Systemt (Promega, Madison, WI, USA).

### Statistical analysis

Data analysis was performed with SPSS 26.0, All data was presented as “mean ± standard error (SEM)”. Significant differences between groups were analyzed using one-way ANOVA. Asterisks signify different significance levels (*P < 0.05, **P < 0.01, and ***P < 0.001).

### Supplementary Information

**Additional file 1: Table S1.** q-PCR primer sequences. **Table S2.** Statistics of downstream data. **Table S3.** Data filtering analysis. **Table S4.** Sequencing data positioning. **Table S5.** RNA-Seq Map Statistics. **Table S6.** Partial lipid metabolism related genes. **Table S7.** SI *vs*. M share gene Target gene.

**Additional file 2: Fig. S1.** Detection of miR-128-3p overexpression interference efficiency Q-PCR primer sequences. **Fig. S2.** Flow cytometry detection. Cell cycle analysis via flow cytometry after overexpression or interference of miR-128-3p.** Fig. S3. **Apoptosis detection. Cell apoptosis assay via flow cytometry after overexpression and interference of miR-128-3p. **Fig. S4. **GO-KEGG enrichment analysis.

## Data Availability

The RNA sequencing data used and analyzed during the current study are available from the NCBI (accession number: PRJNA986221).

## Abbreviations

IMF: Intramuscular fat
miRNA: MicroRNA
mRNA: Messenger RNA
NC group: Blank treatment group
M group: Mimics-treated group
SI group: Inhibitor-treated group
GO: Gene ontology
KEGG: Kyoto encyclopedia of genes and genomes
RT-qPCR: Quantitative real-time PCR
PBS: Phosphate bufered saline
CCK8: Cell Counting Kit-8
IRS2: Insulin receptor substrate 2
IRS1: Insulin receptor substrate 1
Akt: Serine-threonine kinase
LPIN1: Lipin 1
PPARγ: Peroxisome proliferator-activated receptor γ
BMSC: Bone mesenchymal stem cell
FOXO1: Forkhead box protein O1
FOXO4: Forkhead box protein O4
SREBP: Sterol regulatory element-binding protein
ChREBP: Carbohydrate response element binding protein
FABP4: Fatty acid binding protein 4
FASN: Fatty acid synthetase
CEBPA: CCAAT/enhancer-binding protein alpha
BCL2: B-cell lymphoma-2
PCNA: Proliferating Cell Nuclear Antigen
APOA1: Apolipoprotein A1
FDPS: Farnesyl Diphosphate Synthase
MSMO1: Methylsterol Monooxygenase 1
HMGCS1: 3-Hydroxy-3-Methylglutaryl-CoA Synthase 1
GGT5: Gamma-glutamyl transferase 5
TMEM37: Transmembrane protein 37
ASL2: Colletotrichum siamense Argininosuccinate lyase
ACOT13: Acyl-CoA thioesterase 13
GPAM: Glycerol-3-phosphate acyltransferase
HCC: Hepatocellular carcinoma
PIK3R1: Phosphoinositide-3-kinase regulatory subunit 1
MMP9: Matrix metallopeptidase 9
