# Supplementary material for: miR-128-3p inhibits intramuscular adipocytes differentiation in chickens by downregulating FDPS
Source: BMC Genomics. 2023 Sep 12;24:540. doi: 10.1186/s12864-023-09649-y (PMC10496186; doi:10.1186/s12864-023-09649-y)
Supplement: Supplementary file 2 — Additional file 2: Fig. S1. Detection of miR-128-3p overexpression interference efficiency Q-PCR primer sequences. Fig. S2. Flow cytometry detection. Cell cycle analysis via flow cytometry after overexpression or interference of miR-128-3p. Fig. S3. Apoptosis detection. Cell apoptosis assay via flow cytometry after overexpression and interference of miR-128-3p. Fig. S4. GO-KEGG enrichment analysis. [file 12864_2023_9649_MOESM2_ESM.docx]

**
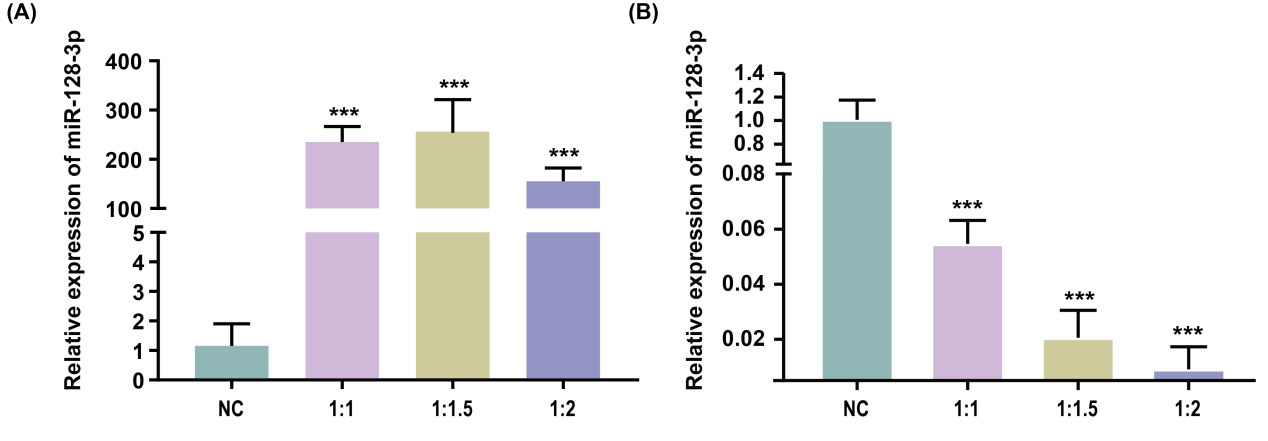
**

**Fig. S1** Detection of miR-128-3p overexpression interference efficiency Q-PCR primer sequences.

1. Detection of overexpression efficiency of miR-128-3p **B.**Detection of interference efficiency of miR-128-3p


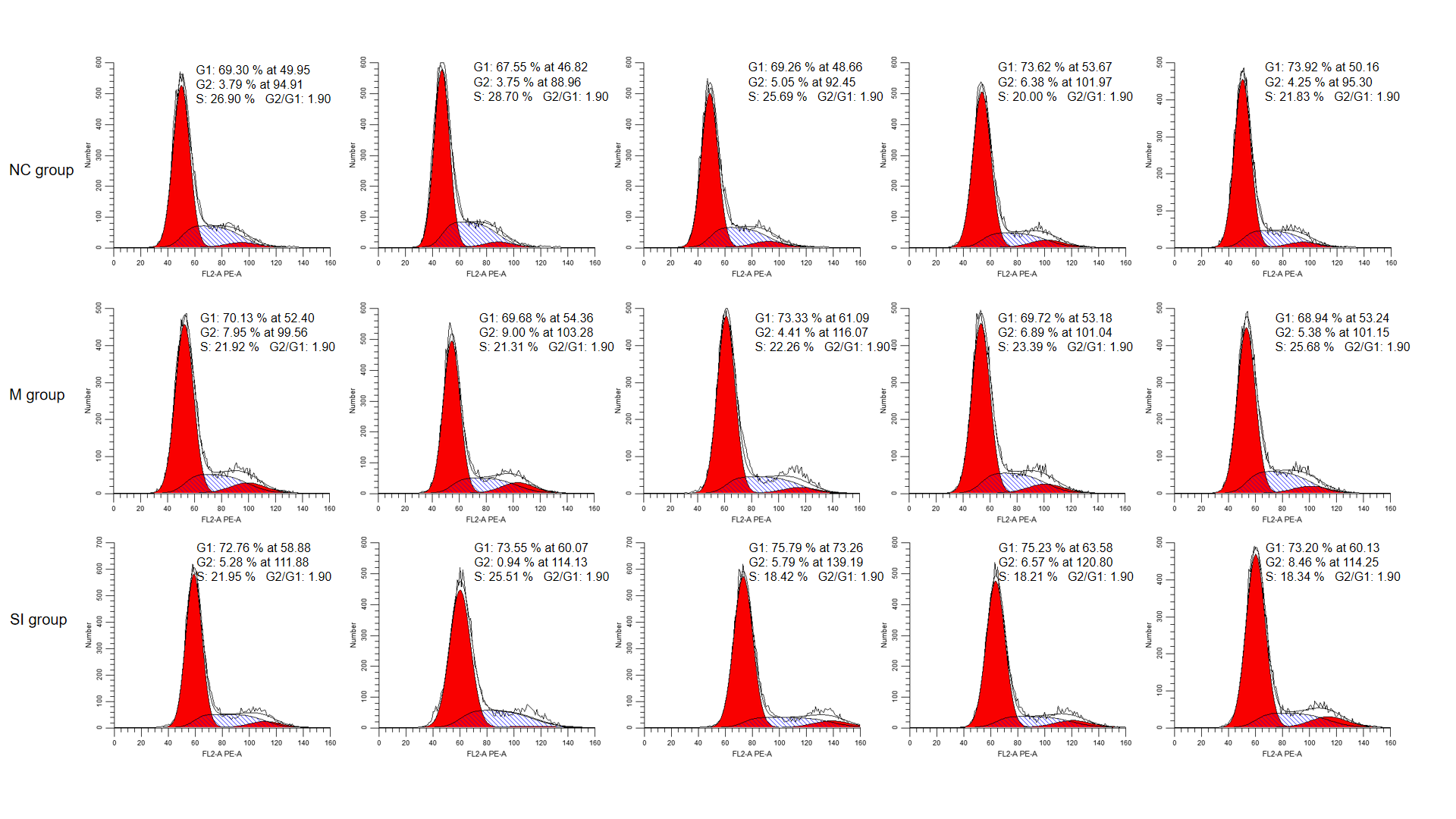


**Fig. S2** Flow cytometry detection. Cell cycle analysis via flow cytometry after overexpression or interference of miR-128-3p. The figure shows NC group, M group (mimics-treated group), and SI group (inhibitor-treated group) from top to bottom (n = 5).


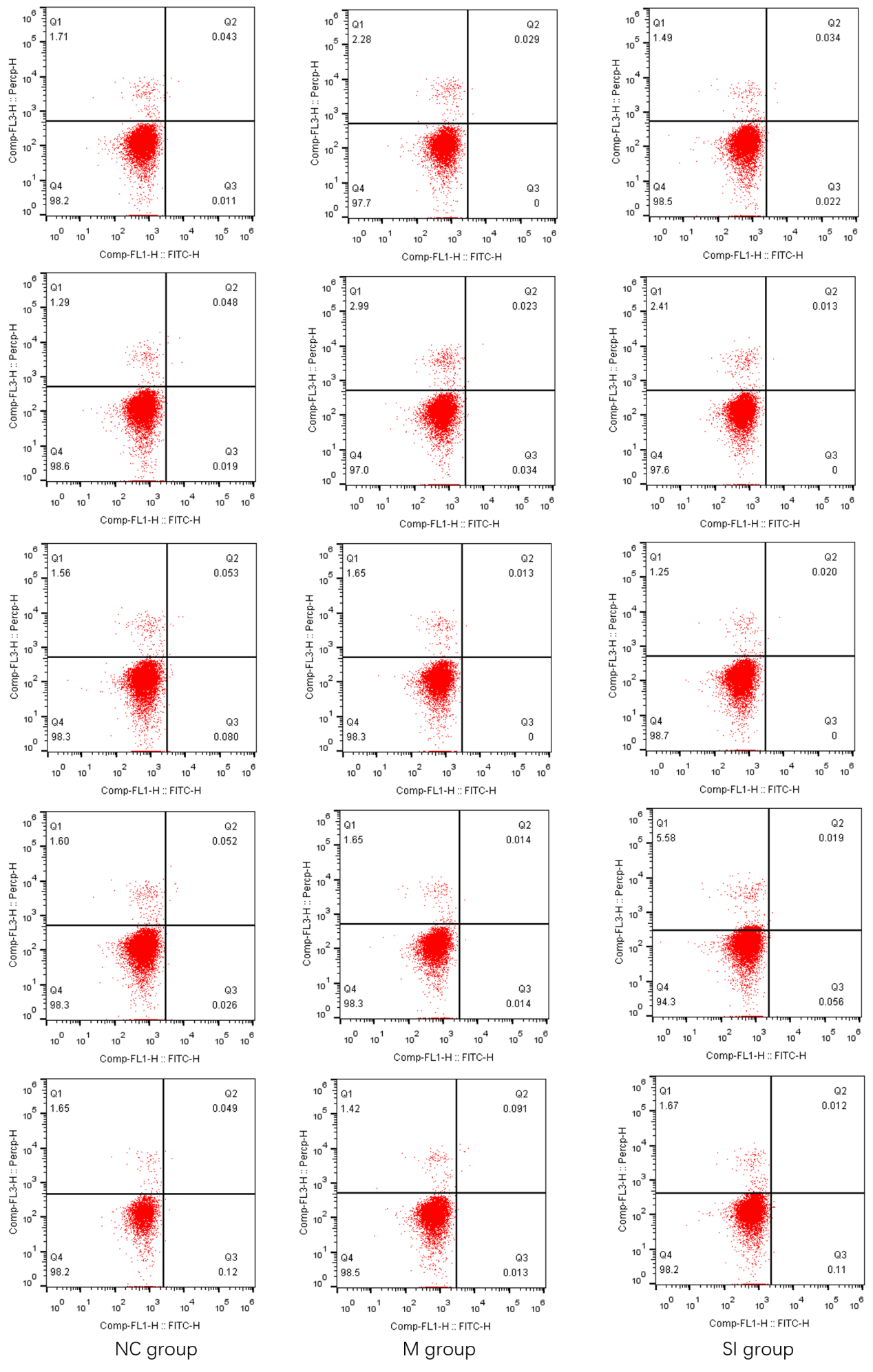


**Fig. S3** Apoptosis detection. Cell apoptosis assay via flow cytometry after overexpression and interference of miR-128-3p.The Fig. shows NC group, M group (mimics-treated group), and SI group (inhibitor-treated group) from left to right (n = 5).


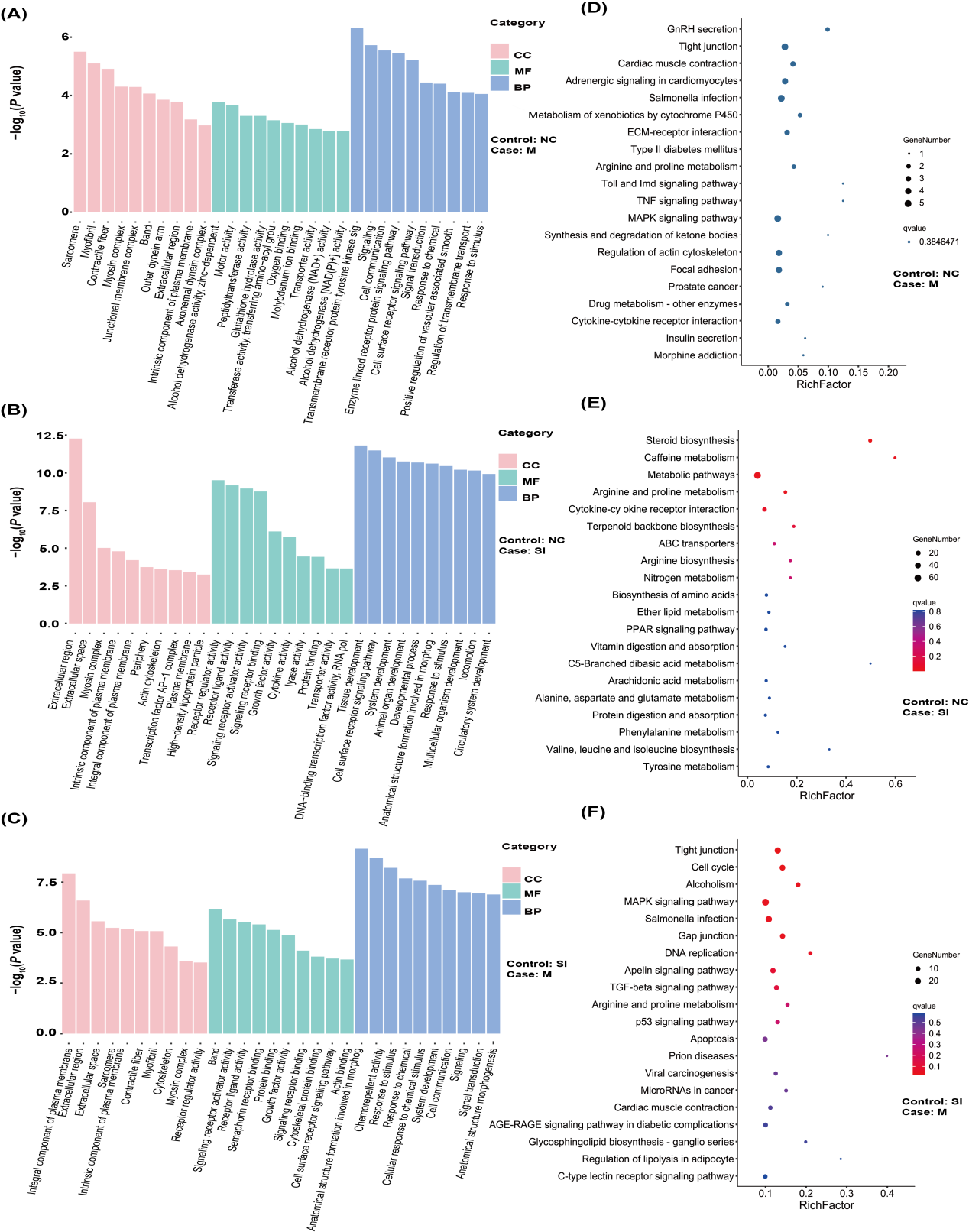


**Fig. S4** GO-KEGG enrichment analysis.**(A-C).** GO enrichment for DEGs from three groups of pairwise comparisons (A: NC vs. M, B: NC vs. Si, and C: M vs. Si). The x-axis presents rich factor of DEGs in a category. The y-axis shows the specific GO term. The top 20 enriched terms are showed according to P-values. (**D-F).** KEGG pathways enrichment for DEGs from three groups of pairwise comparisons (D: NC vs. M, E: NC vs. Si, and F: M vs. Si). The x-axis presents rich factor of DEGs in a category. The y-axis shows the specific pathway. The top 20 enriched pathways are showed according to P-values.
